# Effects of seawater and freshwater challenges on the Gh/Igf system in the saline-tolerant blackchin tilapia *(Sarotherodon melanotheron)*

**DOI:** 10.3389/fendo.2022.976488

**Published:** 2022-10-12

**Authors:** Karl Link, Natallia Shved, Nabil Serrano, Gülfirde Akgül, Antje Caelers, Oliver Faass, Farouhar Mouttet, Oksana Raabe, Helena D’Cotta, Jean-François Baroiller, Elisabeth Eppler

**Affiliations:** ^1^ Institute of Anatomy, University of Zurich, Zürich, Switzerland; ^2^ Institute of Evolutionary Medicine IEM, University of Zürich, Zürich, Switzerland; ^3^ Department of Biomedicine, University of Basel, Basel, Switzerland; ^4^ Institut des Sciences de l’Evolution de Montpellier (ISEM), Université Montpellier, Institut de Recherche pour le Développement (the French National Research Institute for Sustainable Development) (IRD), Ecole Pratique des Hautes Etudes (Practical School of Advanced Studies) (EPHE), Centre National de la Recherche Scientifique (French National Centre for Scientific Research) (CNRS), Unité Mixte de Recherche (Mixed Research Unit) (UMR) 5554, Montpellier, France; ^5^ UMR116-Institut des Sciences de l’Evolution de Montpellier, Centre de Coopération Internationale en Recherche Agronomique pour le Développement, Montpellier, France; ^6^ Institute of Anatomy, University of Bern, Bern, Switzerland

**Keywords:** euryhaline teleost, growth hormone, insulin-like growth factors, cytokines, brain, pituitary, head kidney, spleen

## Abstract

Prolactin (Prl) and growth hormone (Gh) as well as insulin-like growth factor 1 (Igf1) are involved in the physiological adaptation of fish to varying salinities. The Igfs have been also ascribed other physiological roles during development, growth, reproduction and immune regulation. However, the main emphasis in the investigation of osmoregulatory responses has been the endocrine, liver-derived Igf1 route and local regulation within the liver and osmoregulatory organs. Few studies have focused on the impact of salinity alterations on the Gh/Igf-system within the neuroendocrine and immune systems and particularly in a salinity-tolerant species, such as the blackchin tilapia *Sarotherodon melanotheron*. This species is tolerant to hypersalinity and saline variations, but it is confronted by severe climate changes in the Saloum inverse estuary. Here we investigated bidirectional effects of increased salinity followed by its decrease on the gene regulation of *prl*, *gh*, *igf1*, *igf2*, Gh receptor and the tumor-necrosis factor a. A mixed population of sexually mature 14-month old blackchin tilapia adapted to freshwater were first exposed to seawater for one week and then to fresh water for another week. Brain, pituitary, head kidney and spleen were excised at 4 h, 1, 2, 3 and 7 days after both exposures and revealed differential expression patterns. This investigation should give us a better understanding of the role of the Gh/Igf system within the neuroendocrine and immune organs and the impact of bidirectional saline challenges on fish osmoregulation in non-osmoregulatory organs, notably the complex orchestration of growth factors and cytokines.

## Introduction

Mechanisms of physiological adaptations in euryhaline teleost fish species have been mainly identified in a small number of species (including some salmonids, tilapias, but also the killifish, the striped bass and the marine medaka), using individuals maintained under stable conditions or after unidirectional transfers between freshwater and seawater or vice versa (1). Euryhaline fish species can tolerate large changes in salinity by maintaining their hydromineral balance through integrated ion- and water-transporting functions in the major osmoregulatory organs (gills, intestine and kidney) that are responsive to osmosensory and endocrine stimuli (2, 3). However, as reported by Seale and Breves (1) and Blewett et al. (4), in most of these studies the experimental conditions did not consider the highly dynamic dimensions (temporal and spatial scales) of certain aquatic environments such as estuaries, which are essential for understanding the mechanisms of adaptation to increasing environmental instabilities resulting from climate change. Only a few studies in the mummichog and the Mozambique tilapia have investigated the physiological responses under tidally changing salinity regimes. As Seale and Breves (1) demonstrated in their review, the rearing or life history of an individual will influence its osmoregulatory performance: exposure to tidally saline regimes facilitate adaptation to stable hypo- or hyperosmotic environments, maintain seawater-like branchial ionocyte populations and modify the endocrine regulation of osmoregulation towards a local modulation of Prolactin (Prl) signalling (5). The relevance of studies in Mozambique tilapia (*Oreochromis mossambicus*) was emphasized, which demonstrated that fish can maintain osmoregulatory parameters within narrow ranges, and tilapia maintain branchial ionocyte populations in a fashion similar to seawater-acclimated fish (reviewed by 1).

Over the past 40 years, reduced rainfall, longer dry seasons and increased evaporation associated with climate change have dramatically modified the salinity levels of some West African estuaries. This is particularly the case for “inverse” estuaries such as the Sine Saloum in Senegal (6). In this complex habitat, apart from the significant spatio-temporal variations classically observed in estuaries, the Saloum estuary is inverse (with a saline gradient increasing from the embouchure to upstream). Furthermore, at the end of the dry season the salinity can reach 130 ppm, which is more than three-fold the salinity of seawater, in the most upstream areas of the river. These very particular saline spatio-temporal regimes (from 0 to 130 ppm) have affected the biodiversity of this environment. Thus, the estuarine blackchin tilapia species *Sarotherodon melanotheron* is one of the rare fish species that can survive in this environment (7–9). Despite some negative impacts of hypersalinity on its life-history traits (limited growth, reduced size-at-maturity, changing its fecundity), probably associated to an important allocation of energy towards osmoregulation (8), the blackchin tilapia does not migrate during the dry season (10). Moreover, genetically differentiated populations of the same species are also found in Senegal in freshwater, seawater or brackish water. Therefore, besides the Mozambique tilapia, the blackchin tilapia also constitutes an extremely interesting model to better understand the effects of salinity variations, whether they are seasonal (dry and rainy seasons), tidal (in the estuaries) or linked to long-term climatic effects (i.e. hypersalinities in the Saloum inverse estuary), on its physiology. Using different populations living under contrasted saline regimens, recent studies have begun to analyze the mechanisms of the blackchin tilapia’s extraordinary plasticity and adaptation to the significant variations in salinity, both under controlled and natural environments (9, 11–15). In addition, in West Africa, the genus Sarotherodon includes important species for fisheries in estuaries and lagoons but also for aquaculture, e.g. in Senegal and Ivory Coast.

The growth hormone (Gh)/insulin-like growth factor (Igf)1 system has been demonstrated to be involved in several physiological functions in fish (16, 17), particularly in the acclimation to osmotic change (1, 18). In both the Mozambique and blackchin tilapias, the Igfs are involved in osmoregulation (19, 20). In seawater-exposed blackchin tilapia, we have found elevated *igf1*, *igf2* and *ghr1* expressions in gills (20), which is a major osmoregulating organ (1, 21). The inverse was observed after exposure to seawater and recovery with retransfer to freshwater causing decreases in these genes. In contrast, hepatic *igf1*, *igf2* and *ghr1* mRNA decreases after seawater transfer (20). While these studies focused on liver, the source of endocrine Igf1, and osmotic active organs, much less is known with respect to the Gh/Igf system within the brain, pituitary and particularly in immune organs. Prl is essential for freshwater osmoregulation in fish (1, 22), whereas Gh is considered to be involved in seawater adaptation (23, 24). Numerous cell types within the tilapia brain express *igf1* and *igf2*, respectively (25–27). In previous studies, we observed a differential expression of *igf1* and tumor necrosis factor (*tnfa)* in immune organs of tilapia and rainbow trout (28, 29). In order to take into account the large variations in salinity (tidal, seasonal and/or climate change induced) that blackchin tilapia face in the conventional or inverse estuaries in which they live, we have performed here a bidirectional exposure from freshwater to seawater and back to freshwater. We then analyzed the expression changes in *igf1*, *igf2*, and *ghr1* genes in whole brains, pituitaries, spleens and head kidneys as well as the expression changes in *prl* and *gh* genes in the pituitaries. In order to increase our knowledge on the complex orchestration of cytokines and growth factors in the euryhaline blackchin tilapia, we also studied *tnfa* gene expressions in head kidneys and spleens after seawater and freshwater exposures.

## Material and methods

### Animals

The initial broodstock of blackchin tilapia, *Sarotherodon melanotheron heudelotii*, were collected during the rainy season (at a water salinity of 48 ‰) in Kaolack from a wild population in the Saloum estuary in Senegal by Dr. Abdou Mbow (Institut Fondamental d’Afrique Noire, Université Cheikh Anta Diop de Dakar, Senegal), and transferred in 2002 to the experimental facilities of the Centre de Coopération Internationale en Recherche Agronomique pour le Développement (CIRAD) in Montpellier (France) where they were reared in recirculating freshwater at 27 ± 1 °C under a 12 h light/12 h dark cycle. For this study, a mixed batch of sexually mature 14-month-old fish from a 4^th^ generation progeny of the fish stocked in the CIRAD facilities was used. All the experimental procedures took place in CIRAD facilities under the Laboratory agreement for animal experimentation number A-34-172-24 and the author’s personal authorization for animal experimentation N° 35-15, both authorised by the French Government.

### Seawater and freshwater experiments

In order to avoid a handling stress during the freshwater to seawater or the seawater to freshwater experiments (30), the fish were kept in the freshwater aquaria, and half of the water was changed by aspiration and replaced by saline water prepared from a stock solution of 100 ‰ made from OCEAN INSTANT^®^ salts at a rate of 0.5 ‰ per min until the experimental seawater salinity of 35 ‰ was reached, as previously described (20). Salinity was monitored daily. Similarly, for the seawater to freshwater experiment, seawater was changed by aspiration and replaced by freshwater at the same exchange rate until the 0 ‰ salinity was reached. In order to standardize the stress in all fish groups, at each salinity change (freshwater to seawater or seawater to freshwater), a freshwater change was also conducted in the freshwater control group, i.e., half of the water was changed by aspiration and replaced by freshwater.

### Fish sampling and tissue preparation

One hundred and ten individuals were maintained in 11 freshwater aquaria of 40 l each, with 10 fish per aquarium under a 12 h/12 h light/dark cycle. Each aquarium was equipped with an individual filter, a temperature-controlled heater (27 ± 1 °C) and an aerator. Fish were fed to satiation three times a day with BIOMAR-SA food (BIOMAR-S.A., Nersac, France) until 24 h before sampling. Ten aquaria were used for the salinity challenges of the experimental fish: five aquaria were used for the five seawater sampling time points. In the other five aquaria, the seawater to freshwater retransfer was then performed after one week. The 11^th^ aquarium was the freshwater control batch. For each sampling time point (4 h, 1, 2, 3 and 7 days after water exchange), all the ten fish from an aquarium were anaesthetized together, sacrificed and samples collected. For that purpose, one dose of 2-phenoxyethanol (Sigma, St. Louis, MO, USA) was introduced directly into the water of one aquaria to reach a dose of 2 ml/l. At each sampling time point, fish were weighted and measured. Both experimental groups were similar in size, with an average length of 11.6 ± 0.6 cm and weight of 26.2 ± 5.0 g in the seawater group and an average length of 11.8 ± 0.7 cm, and weight of 27.4 ± 4.2 g in the freshwater control group. Intact brain, pituitary, head kidney and spleen were excised and transferred into 1.5 ml of RNAlater™ (Ambion, Austin, TX, USA) for RNA preservation, kept at 4°C overnight to allow RNAse-inhibitors to act and then stored at -20°C until RNA extraction.

### Design of primers and probes for quantitative real-timePCR

ZF gene and protein nomenclature rules were followed (31). TaqMan real-time PCR assays were created (**Table 1**) based on mRNA sequences of tilapia species, i.e., *Sarotherodon melanotheron prl* (32), *Oreochromis mossambicus igf1* (33), *igf2* (34), and RNA ribosomal 18s (*rna18s)* (35), and *Oreochromis niloticus gh* (36), *ghr1* (37), *tnfa* (38), *actin beta (actb)* (39), and *eukaryotic translation elongation factor 1a (eef1a)* (40), as previously described (26, 28, 41–43). Taqman probes were dually labelled at the 5’ end with the fluorescent reporter dye FAM and at the 3’ end with the quencher TAMRA. Primer accuracy was verified as published for *igf1*, *igf2*, *gh* and *actb* (25, 26), and *ghr1*, *tnfa* and *eef1a* (20). A comparison of three different reference genes (*actb, eef1a, and rna18s*) demonstrated that *actb* was most stable across these experimental groups as previously evaluated for liver and osmoregulatory organs (20).

**Table 1:** Quantitative real-time PCR assays used in this study.

Gene	Primers: F - forward, R - reverse	Probe: FAM-TAMRA	Amplicon(bp)	Accession number
*gh*	F: TCGACAAACACGAGACGCA R: CCCAGGACTCAACCAGTCCA	CGCAGCTCGGTCCTGAAGCTG	75	A08993
*ghr1*	F: CAGACTTCTACGCTCAGGTC R: CTGGATTCTGAGTTGCTGTC	CAACACCACACCACCAGTTGGC	80	AY973232
*igf1*	F: GTTTGTCTGTGGAGAGCGAGG R: GAAGCAGCACTCGTCCACG	CAATAAACCAACAGGCTATGGCCCCAG	97	Y10830
*igf2*	F: CAGTTTGTCTGTGAAGACAGAGGC R: CTCCTCTACGATCCCACGGG	CGTCGGTTGTTACCCCTGCTGGTTG	93	AF033801
*prl*	F: CATGACAAGATCACCAAGT R: CTGTCAATCTTGTGAGAGTC	CAACTTCAACTTTCTGCTGTCCTG	77	GQ359823
*tnfa*	F: GAGTGGAGGAATGGTCAAGG R: GTGTGGGATGATGATTTTATTGG	TGAAGCCGCCCTGAGCAAACGC	80	AY428948
*actb*	F: GTATGTGCAAGGCCGGATTC R: CCATCACACCCTGATGCCTG	CGACGCCCCTCGTGCTGTCTACC	90	AY116536
*eef1a*	F: CGAGGAAATCACCAAGGAAG R: AGATGGGGACGAAGGCAAC	CCTACATCAAGAAGATCGGCTACA	84	AB075952
*rna18s*	F: GGTTGCAAAGCTGAAACTTAAAGG R: TTCCCGTGTTGAGTCAAATTAAGC	ACTCCTGGTGGTGCCCTTCCGTCA	85	AF497908

bp, base pairs.

### Real-time qPCR

**Figure 1:**
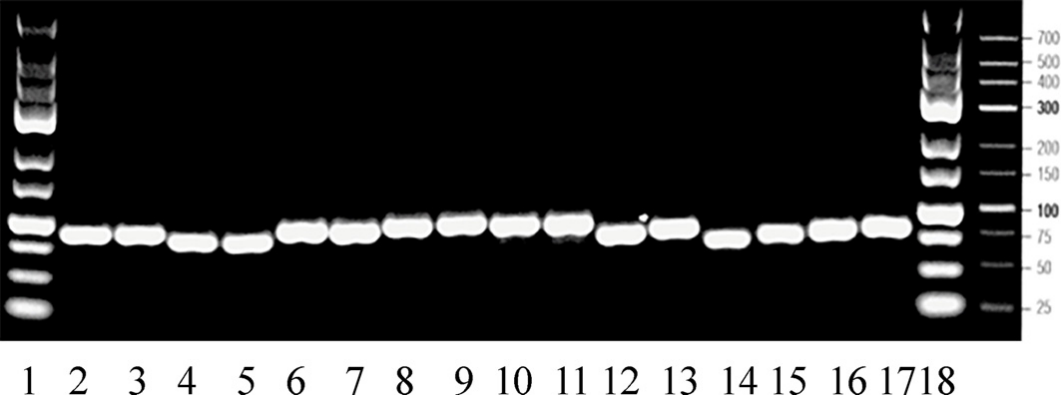
Gel electrophoresis of real-time PCR amplification products at their expected sizes between 75-97 bps (compare Table 1). Lane 1: 25 bp molecular weight marker, lanes 2-3: *actb*, lanes 4-5: *rna18s*, lanes 6-7: *igf1*, lanes 8-9: *igf2*, lanes 10-11: *ghr1*, lanes 12-13: *gh*, lanes 14-15: *prl*, lanes 16-17: *tnf*a, lane 18: 25 bp molecular weight marker.

Total RNA was extracted using the TRIzol™reagent (Invitrogen, Merelbeke, Belgium), chloroform, isopropanol and ethanol, re-suspended in RNase free water and photospectrometrically quantified. Total RNA was treated with 1 U/μl of RQ1 RNAse-free DNAse (Catalys AG, Switzerland). Single-stranded cDNA was synthesized from 800 ng of total RNA using 1 x TaqMan^®^ RT Buffer, MgCl_2_ (5.5 mM), 1.25 U/μl of MuLV reverse transcriptase, 2.5 μM of random hexamer primers, 0.4 U/μl ribonuclease inhibitor, and 500 μM of each dNTP (Applied Biosystems, Rotkreuz, Switzerland) for 10 min at 25 ˚C, 30 min at 48 ˚C and 5 min at 95 ˚C. Then 2 μl cDNA obtained from 10 ng/μl total RNA were subjected, in duplicate, to real-time PCR using an Absolute™ QPCR low ROX Mix (ABgene, UK) including Thermo-Start^®^ DNA Polymerase, 300 nM of each primer and 150 nM of the fluorogenic probe. Amplification was performed with a total reaction volume of 10 μl in a MicroAmp Fast Optical 96-well reaction plate (Applied Biosystems). The reactions were run on the ABI 7500 Fast real-time PCR System (Applied Biosystems) under the following conditions: 15 min at 95 ˚C for enzyme activation followed by 40 cycles of 95 ˚C for 15 s and 60 ˚C for 1 min. The PCR amplification products were verified on gel electrophoresis (**Figure 1**) using a GeneRuler 25 bp molecular weight marker and loading buffer (both Fermentas, Le Mont sur Lausanne, Switzerland).

### Relative quantification of salinity response

The relative gene expression model was used to evaluate the n-fold changes in mRNA expression between experimental and control fish for each sampling time point using the ΔΔCT method (44) as established (20, 28, 29, 41–43, 45). Prior to analysis, real-time PCR assays were validated by plotting CT values against the logarithms of the dilution factors. Slope was determined and the correlation of efficiencies between compared assays confirmed as described for *igf1*, *gh* and *actb* (26, 46). Relative gene expression ratios (R) between treated and control groups were calculated using the formula: R = 2^-ΔΔCT^ with ΔCT = CT (target gene) - CT (reference gene) and ΔΔCT = ΔCT (treated group) - ΔCT (untreated control group). Thus, experimentally induced changes are presented as *n*-fold differences relative to the corresponding untreated control set to 1. Significant differences between treated and untreated groups are designated by asterisks. Significant differences between time points are designated by different small letters. Statistical significance was calculated using the Mann-Whitney rank sum test. Statistical analyzes were performed with GraphPad Prism^©^ 5 (Graphpad Software, Inc., San Diego, CA, USA).

## Results

### Effects of seawater and freshwater exposure on whole brain *ghr1, igf1*, and *igf2*

**Figure 2:**
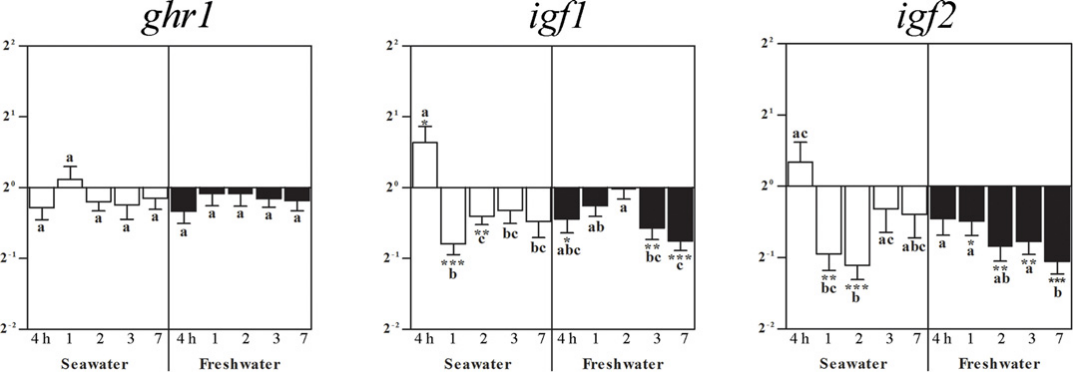
Effects of salinity on brain *ghr1*, *igf1*, and *igf2* gene expressions. Relative changes (log_2_, y-axis) of mRNA expression at 4 h, 1, 2, 3 and 7 days (x-axis) after exposure to seawater (white columns) and freshwater (black columns). Bars represent mean ratios between treated and control group ± SEM and asterisks show significant differences: * *p* < 0.05; ** *p* < 0.005; *** *p* < 0.0005. Bars within treatment groups that do not share any letters are significantly different from each other with p < 0.05, e.g., *igf1* seawater expression between 4 h and 1 day, 2, 3 days and 7 days are significantly different, but between 1 day or 2 days, and 3 or 7 days are not significantly different.

In brain, *ghr1* gene expression was not altered by seawater nor by freshwater (**Figure 2**). Brain *igf1* mRNA elevated 4 h after exposition to seawater (**Figure 2**). At 1 and 2 days after seawater exposure, *igf1* and *igf2* genes significantly decreased (**Figure 2**). After 1 week in seawater, restitution of freshwater lowered *igf1*mRNA after 4 h, and after 3 and 7 days. Gene expression of *igf2* mRNA decreased one day after freshwater exposure and persisted at a lower level until the end of the study (**Figure 2**).

### Effects of seawater and freshwater exposure on pituitary *prl*, *gh*, *ghr1*, *igf1*, and *igf2*

**Figure 3:**
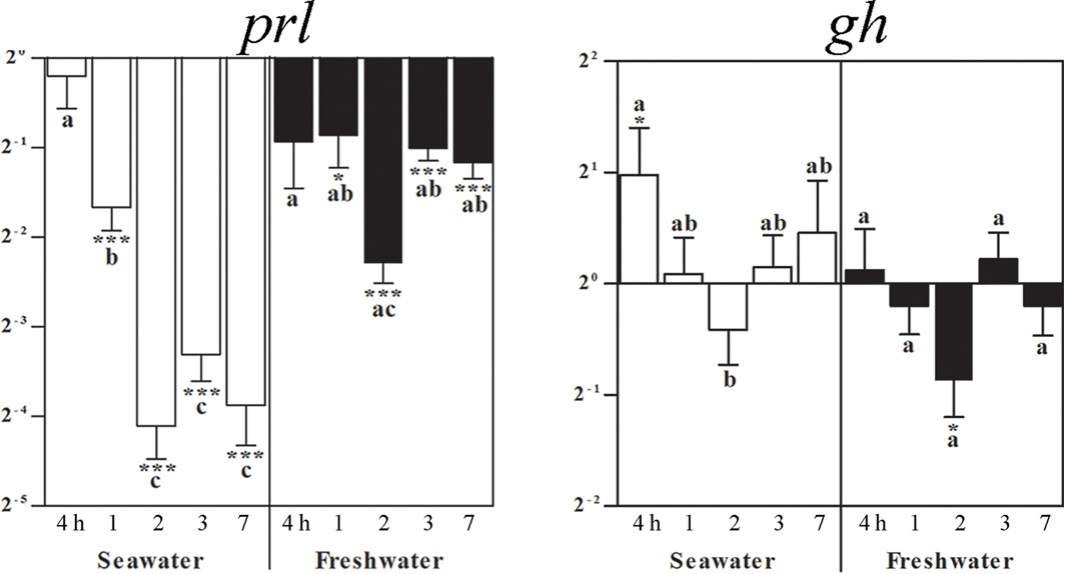
Effects of a seawater and freshwater exposure on pituitary *prl* and *gh* gene expressions. Relative changes (log 2, y-axis) of mRNA expression at 4 h, 1, 2, 3 and 7 days (x-axis) after exposure to seawater (white columns) and freshwater (black columns). Bars represent mean ratios between treated and control group ± SEM and asterisks show significant differences: * *p* < 0.05; *** *p* < 0.0005. Bars within treatment groups that do not share any letters are significantly different from each other: *p* < 0.05., e.g., *prl* seawater expression between 4 h and 1 day, 2, 3 or 7 days are significantly different, but between 2 and 3 or 7 days are not significantly different. :Starting 1 day after return of freshwater, a significant difference to untreated freshwater controls was observed, but not between time points.

From the first post-experimental day on, *prl* mRNA lowered during the entire seawater period. After return of freshwater, experimental group levels raised back to those of freshwater controls to become significantly lower again after 1 day for the entire freshwater period (**Figure 3**). At 4 h after exposure to seawater, *gh* mRNA increased and returned to control levels. At 2 days after freshwater exposure, *gh* mRNA decreased and then returned once again to the control level (**Figure 3**).

**Figure 4:**
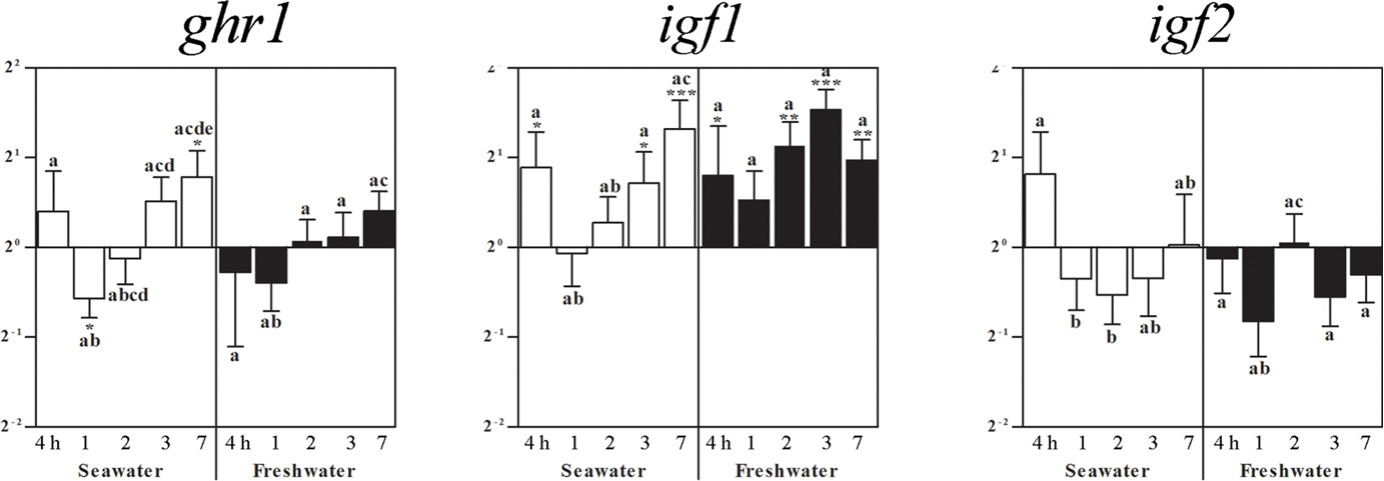
Effects of exposure to seawater and freshwater on pituitary *ghr1*, *igf1*, and *igf2* gene expressions. Relative changes (log_2_, y-axis) of mRNA expression at 4 h, 1, 2, 3 and 7 days (x-axis) after exposure to seawater (white columns) and freshwater (black columns). Bars represent mean ratios between treated and control group ± SEM and asterisks show significant differences: * *p* < 0.05; ** *p* < 0.005; *** *p* < 0.0005. Bars within treatment groups that do not share any letters are significantly different from each other: *p* < 0.05, e.g., *igf2* seawater expression between 4 h and 1 or 2 days are significantly different, but between 4 h and 3 or 7 days and between 1 or 2 days and 3 or 7 days are not significantly different.

Exposure to seawater lowered the *ghr1* gene expression in the pituitary after 1 day, which was compensated to elevated levels after 7 days (**Figure 4**). Restitution of freshwater did not significantly alter *ghr1* mRNA as compared to the control fish (set as 1) (**Figure 4**). After exposure to seawater, pituitary *igf1*mRNA was elevated at 4 h, 3 and 7 days (**Figure 4**). The maintenance for 7 days in seawater followed by the exposure to freshwater resulted in elevated *igf1* mRNA levels already after 4 hours, which remained elevated for the rest of the experimental period. The *igf2* mRNA levels did not significantly differ from those of the control fish, but differed between time points (**Figure 4**).

### Effects of seawater and freshwater exposure on *ghr1*, *igf1*, *igf2*, and *tnfa* in head kidney

**Figure 5:**
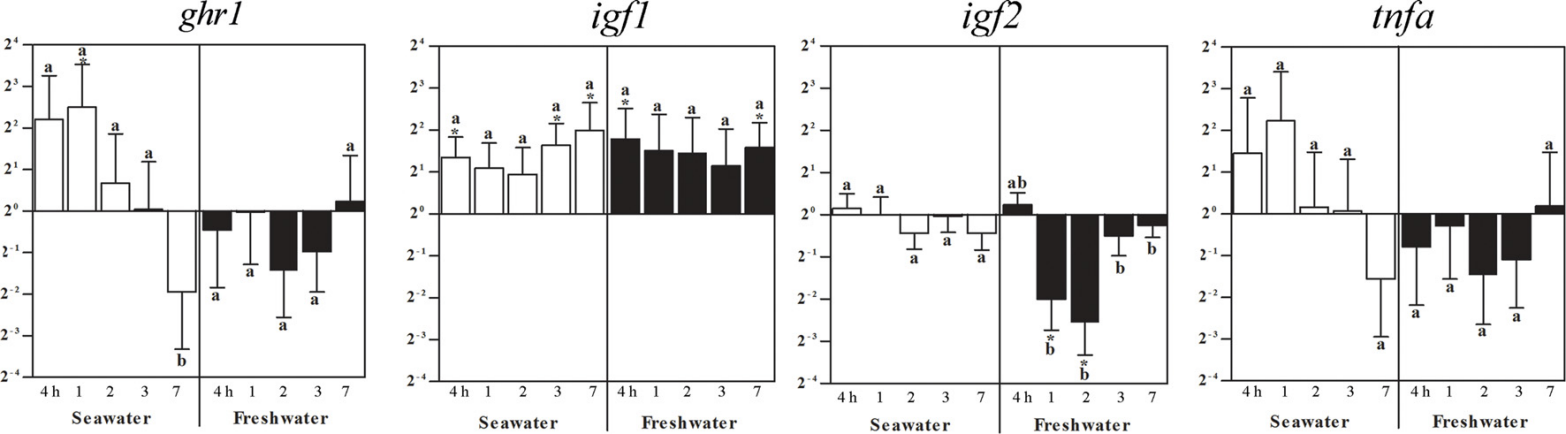
Effects of a seawater and freshwater exposure on the head kidney *ghr1*, *igf1*, *igf2, and tnfa* gene expressions. Relative changes (log_2_, y-axis) of mRNA expression at 4 h, 1, 2, 3 and 7 days (x-axis) after exposure to seawater (white columns) and freshwater (black columns). Bars represent mean ratios between treated and control group ± SEM and asterisks show significant differences: * *p* < 0.05; *** p< 0.0005. Bars within treatment groups that do not share any letters are significantly different from each other: p < 0.05, e.g., *ghr1* seawater expression between 4 hours, 1, 2 or 3 days and 7 days are significantly different, but between 4 h and 1 day, 2 or 3 days are not significantly different.

In the head kidney, *ghr1* expression was elevated 1 day after seawater challenge while no significant changes compared to the freshwater controls (set as 1) occurred at the other times points. After 7 days in seawater, however, the *ghr* gene expression was lower than at all other time points, also after restitution of freshwater (**Figure 5**). Four hours after exposure to seawater, *igf1* mRNA increased and persisted with a tendency to elevation becoming significant again after 3 and 7 days (**Figure 5**). After the return to freshwater, *igf1* gene expression was similarly elevated throughout the entire treatment period (**Figure 5**). While at the other time points no significant changes in *igf2* expression were observed, it decreased at 1 and 2 days after return to freshwater and recovered after 3 days. No significant changes occurred for *tnfa* (**Figure 5**).

### Effects of seawater and freshwater exposure on *ghr1*, *igf1*, *igf2*, and *tnfa*, in spleen

**Figure 6:**
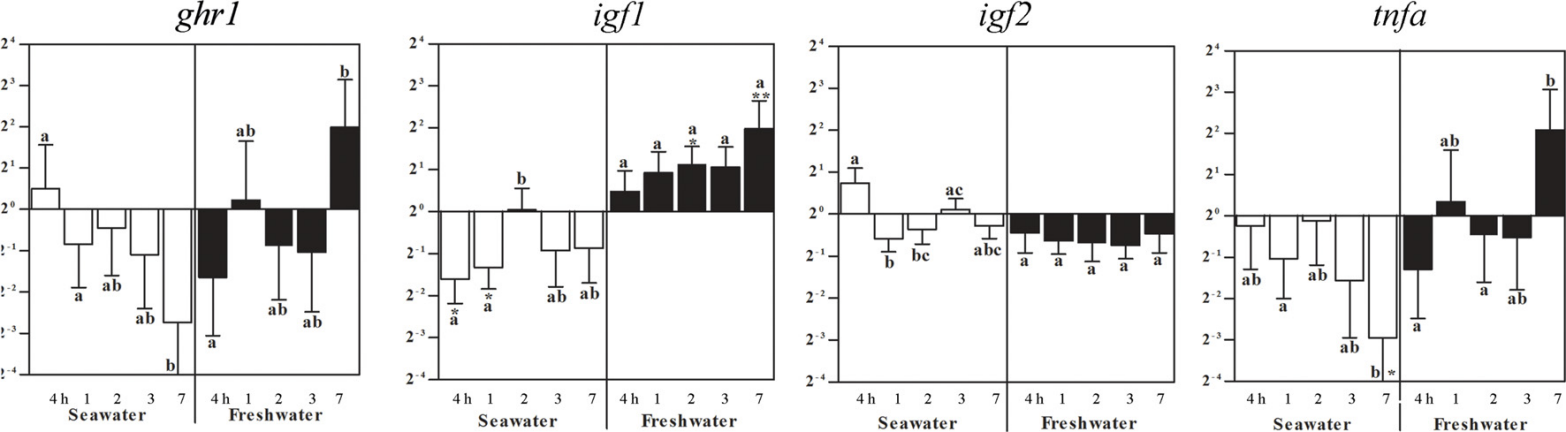
Effects of a seawater and freshwater exposures on the spleen *ghr1*, *igf1*, and *igf2* and *tnfa* gene expressions. Relative changes (log_2_, y-axis) of mRNA expression at 4 h, 1, 2, 3 and 7 days (x-axis) after exposure to seawater (white columns) and restitution of freshwater (black columns). Bars represent mean ratios between treated and control groups ± SEM and asterisks show significant differences: * *p* < 0.05; ** *p* < 0.005. Bars within treatment groups that do not share any letters are significantly different from each other: *p* < 0.05, e.g., *tnfa* seawater expression after 7 days was significantly lower than in untreated controls, and differed to fish after 1 day in seawater, but not after 4 h, 2 or 3 days.

In the spleen, *ghr1* and *igf2* expressions were not significantly different to control levels (set as 1), but differed between time points (**Figure 6**). After exposure to seawater, *igf1* mRNA decreased after 4 h and 1 day, which reversed by the return of freshwater to elevated levels after 2 and 7 days (**Figure 6**). After exposure to seawater, *tnfa* gene expression differed between the time points until it significantly decreased after 7 days. After return of freshwater, no significant differences to the control fish levels occurred, but between time points (**Figure 6**).

## Discussion

Osmoregulation is an essential physiological adaptation required for health and reproduction of numerous euryhaline fish species (23, 47–51). The blackchin tilapia is an euryhaline species, which has evolved particular physiological adaptations to drastic salinity changes (52). Nevertheless, climate changes appear to severely impact fish physiology (53). Salinity changes occur in lakes, rivers and estuarines’ ecosystems in West Africa where increased salinities, due to the combined effects of reduced freshwater inflow and water evaporation on the one hand (12), and on the other hand seasonal rainfalls decrease water salinity (9). Increasing drought periods particularly affect some Sahelian estuaries, which is the case in the Sine Saloum River of Senegal (6, 8, 11, 13).

### Effects of seawater and freshwater exposure on teleost Gh/Igf-system

Environmental salinity exerts pronounced effects on somatic growth and on the Gh/Igf1 axis of the juvenile Mozambique tilapias (5, 54). To date, main emphasis on osmoregulatory responses of the Gh/Igf1 system has been laid on the endocrine, liver-derived Igf1 route (5, 55, 56), and on local regulation within the liver and osmoregulatory organs (20, 55–58), as well as *gh* and *prl* expressions in the pituitary (5, 54). Nevertheless, environmental alterations of the Gh/Igf-system also impact other physiological systems including the immune system [for reviews see: (59– 64)]. We here explored the effects of seawater and freshwater exposures on the *igf1*, *igf2* and *ghr1* genes in brain, pituitary and immune organs and additionally, *gh* and *prl* genes in the pituitary and *tnfa* gene in the immune organs head kidney and spleen.

In our preceding study, we found elevated expressions of the *igf1*, *igf2* and *ghr1* genes in the gills of the blackchin tilapia after exposure to seawater and in part, after returning to freshwater (20). Likewise, *ghr1* expression was upregulated in freshwater gills in wild sockeye salmon (*Oncorhynchus nerka*) (65). Thus, there exists evidence for a differential involvement of the Gh/Igf-system in osmoregulation at extrahepatic sites, but the data set is limited. Most recently, the Gh/Igf1 axis together with cortisol were found to be strongly involved in seawater adaptation and osmoregulation in Atlantic salmon (*Salmo salar*) as observed for muscle tissue (66). A particular local role of *igf1* was proposed for fast signalling on the salt secretory system in euryhaline teleosts in response to salinity stress (58). Both *igf1* mRNA and Igf1 peptide were detected in the majority of ionocytes of developing and adult Nile tilapia (27). These gills ionocytes are in combination with prolactin signalling crucial for salinity adaptation in euryhaline fish (1). In the present study, we focused on brain, pituitary and the immune organs head kidney and spleen.

### Effects of seawater and re-exposure to freshwater on brain *ghr1, igf1* and *igf2*

Four hours after exposure to seawater, brain *igf1* mRNA was elevated, but subsequently both, *igf1* and *igf2* mRNA levels decreased from day 1 onwards, and levels remained low after return of freshwater, while *ghr1* mRNA was not affected. In brain neurons of juvenile and, to a lesser extent adult Nile tilapia, *igf1* expression was detected in different regions including cerebellum (27, 67), periventricular zone and optic tectum (41). In our preceding study, transfer to seawater lowered *igf1* mRNA in the intestine at 4 h, which increased in a stepwise manner becoming significant at 2 days. After exposure to freshwater, intestinal *igf1* gene was elevated during the entire experimental period (20). Specific alterations upon hypoosmotic stress such as disturbed gut-brain neurotransmission, impaired intestinal barrier integrity, and inflammatory reactions were recently described in the yellowfin seabream (*Acanthopagrus latus*), including changes in the tight junction protein ZO-2 and interleukin 1 beta (*il1b*) (68). An evolutionary well-conserved role of Igf1 was suggested for paracellular transport and organization of epithelia such as formation of tight junctions (e.g., 69–71). Similarly, the importance of Igf1 for intercellular junctions and tissue architecture was proposed for liver (72, 73), the main source of endocrine Igf1. In addition, in human lymph nodes, our group had detected Igf1 immunoreactivity in high-endothelial venules, site of lymphodiapedesis, which we interpreted as particular relevance of Igf1 for maintenance and restoration of cellular integrity and tissue organization (74). These rudimentary data might point to a role of Igf1 also in the brain-gut-axis to cope with alterations of the water salinity environment *via* intercellular connections.

Significantly lower *igf2* gene expressions compared to control fish were observed in the present study after seawater and freshwater exposure exclusively in the brain. In adult Mozambique tilapia, *igf2* expression was widespread in neurons, meninges and choroid plexus of diverse brain regions, which was interpreted as a role in the differentiation, maintenance and regeneration of neurons, particularly for the continued post-embryonic life-long brain growth in many teleost fishes (75). However, this was even lower than *igf1* expression as measured by absolute transcript quantification (26). In the present study, there was a general trend to lower levels of *igf2* compared to control fish in brain, which was also observed in the other organs after either treatment. Lower *igf2* in contrast to *igf1* gene expressions were also observed in seabream juveniles *(Sparus aurata)* one hour after stress conditions of salinity, temperature and ammonia, whereby *igf1* was emphasized to be more under control of Gh (76). Organ specific alterations of *igf1* and *igf2* after Gh treatment were observed in rainbow trout (*Oncorhynchus mykiss*) (77), Nile tilapia pituitary and immune organs (28, 43).

### Effects of seawater exposure and freshwater re-exposure on the pituitary *prl, gh, ghr1, igf1* and *igf2* transcripts

The adenohypophyseal hormones Prl and Gh are involved in the physiological adaptation to varying salinities (5, 20, 49, 55, 56, 58, 66, 78). In the present study, we observed a decrease in *prl* mRNA levels in the blackchin tilapia starting from the first post-experimental day after seawater exposure and during the entire seawater period, which back in freshwater raised to baseline control levels. This is well in line with the observation in isolated Prl cells that the salinity acclimation history of fish influences the osmotic responsiveness. For instance, baseline gene expressions of *prl177* and *prl188* were 30-fold higher in Prl cells isolated from Mozambique tilapia acclimated to freshwater versus fish acclimated to seawater. These Prl cells were less responsive to hyposmotic stimulation with regard to prl mRNA levels (79), which might explain, even with respect to species differences, that the freshwater-acclimated blackchin tilapia in our study did not strongly react after their return from seawater back to freshwater. Thus, differences in responses of Prls and *prls* to extracellular osmolality between tilapia species may be linked, at least in part, to the observed inter-species difference in salinity tolerance as reviewed by Seale and co-workers (2020).

Return of freshwater elevated *prl* gene expressions after 4 h and lowered them again for 7 days. Most recently, Prl has been proposed to promote freshwater acclimation in mummichogs by orchestrating the expression of solute transporters/channels, water channels, and tight-junction proteins. In freshwater-type ionocytes, ovine Prl stimulated the expression of Na^+^/Cl^-^ cotransporter 2 and aquaporin 3, whereas it lowered gene expressions in seawater-type ionocytes of the Na^+^/K^+^/2Cl^-^ cotransporter 1, cystic fibrosis transmembrane regulator 1, and claudin 10f (80). In mammals, Prl is the central hormone to promote during pregnancy mammary cell proliferation and differentiation to prepare for lactation. From an evolutionary aspect, secretion of a mineral-enriched solution from skin glands to protect soil-laid eggs from heat and dryness was described for amphibians and reptiles (81–83). Prl is a pleiotropic hormone, which was found to be produced also at extrapituitary sites and to act on the brain in manifold ways (for review: 84).

In contrast to *prl* mRNA, seawater challenge increased *gh* mRNA at 4 h whereas freshwater exposure decreased it. Our results are well in line with higher *gh* expressions found in seawater than in freshwater exposed Mozambique tilapia (5). The data regarding Gh levels under salinity changes are, however, contradictory, whereby several studies proposed *gh* mRNA as a better indicator of growth than Gh serum levels (5, 54, 85). As also demonstrated in Mozambique tilapia individuals under controlled conditions (86), populations of blackchin tilapia living in seawater had a better growth rate than those living in freshwater (8, 11). Individual growth rates in that study were calculated by the ratio between fish length and the actual fish age in years estimated by counting the macro-increments on whole otoliths according to the validated method developed by Panfili and co-workers (2004). In contrast, those living under hypersaline conditions (Saloum estuary) exhibited the lowest growth rate, probably reflecting the energy costs of adapting to extreme salinities (11). In the pituitary of these individuals, *gh* expression was higher in populations of blackchin tilapias adapted to seawater than in those living in freshwater, brackish or hypersaline environments (11). In that population study, *gh* expression levels seemed to be better related to growth rates in each environment than to salinities. Nevertheless, important variations of both *prl1* and *gh* gene expressions were reported between individuals for each population.

In the preceding study on osmoregulatory organs (20), salinity challenges did not cause a significant change in renal *igf1* mRNA. In the gills, *igf1* mRNA was decreased after 2 days in seawater to become elevated after 3 and 7 days. We found *igf1* mRNA expression in the blackchin tilapia pituitary elevated after 4 h of exposure to seawater and then again after 3 and 7 days, remaining high when re-exposed to freshwater, while *igf2* mRNA was not altered. Pituitary *ghr1* in seawater followed somewhat the trend of *igf1* with levels increasing only after 3 days, but upon freshwater exposure, *ghr* levels differed only slightly between groups. Similarly, in the pituitary of seawater-acclimated Mozambique tilapia, *ghr1* and *igf1* increased, but with no difference in *gh* gene suggesting that local Prl-releasing peptide and dopamine 2 receptor may control and regulate *prl* gene expression in the pituitary (87). Nevertheless, in those pituitary hormone cell types supposed to be differentially involved in a broad range of physiological actions including osmoregulation (88), *igf1* gene or Igf1 peptide were not detected in freshwater-reared Nile tilapia, i.e., the somatolactin cells in the pars intermedia and the prolactin cells in the rostral pars distalis (89).

On the contrary, pronounced Igf1 immunoreactivity in pituitary adrenocorticotrophic hormone (ACTH) cells was well preserved throughout evolution (61, 62), which was interpreted as a particular need of ACTH cells for maintenance and restoration (89), also under salinity stress for a healthy and functioning immune axis (87). Most recently, the importance of ACTH in electrolyte homeostasis *via* the mineralocorticoid 2 receptor was emphasized (90).

### Effects of seawater and re-exposure to freshwater on *ghr1*, *igf1*, *igf2*, and *tnfa* gene expressions in head kidney and spleen

Alterations of natural salinities have been proposed to impair the immune system of fish (91). Increased salinity in Nile tilapia (92), did not significantly affect any immune system assays, whereas decreased salinity was accompanied by lymphopenia, neutrophilia and monocytosis in the peripheral blood without modifying total cell volume, plasma protein or cortisol levels. Phagocytosis was increased without change of the phagocytic index. Transforming growth factor beta (*tgfb*) transcription increased, but transcription of *il1b* did not change. In that study, acute salinity changes appeared to trigger reactive dysregulation of the immune response in tilapia, which in combination with additional stressors could make fish more susceptible to infectious diseases (92). For instance, in the intestine of *Amyloodinum ocellatum*-infected juvenile European sea bass (*Dicentrarchus labrax*), cytokines including *tnfa* increased, which was proposed to help fish to cope with the infection (93). Interestingly, in head kidney and spleen of *Yersinia ruckeri*-challenged rainbow trout *(Oncorhynchus mykiss)*, *igf1* and *tnfa* were simultaneously elevated while again *igf2* expression was, if at all affected, lowered (29).

Very few data exist on the local role of Igf1 and even less for Igf2, in fish immune organs generally. In the present study, 4 h after exposure to seawater, *igf1* expression in the head kidney increased and remained elevated until the end of the experiment, and this was the case also after return of freshwater. No significant changes were observed in *tnfa* expression, whereby a similar tendency to elevated levels in seawater and lowered levels in freshwater was observed and likewise for *ghr1*. In spleen, *tnfa* expression was lower only after 7 days in seawater, but there was again a similar tendency to lower levels in seawater seen equally for *ghr1*. After exposure to seawater, *igf1* mRNA decreased, which reversed when returned to freshwater reaching levels that became significantly elevated after 2 and 7 days.

The role of the Gh/Igf-system has been under debate for the fish immune system for a long time (reviewed by 64). In *gh*-overexpressing Mozambique tilapia, *igf1* expression was lower than in the spleen of control fish (43). Recently, a differential role of cytokines and the Gh/Igf axis has been emphasized from *in vitro* studies in Atlantic salmon (94) and *in vivo* studies in zebrafish (95).

The present study contributes tissue specific effects on gene expressions of the *igfs* and *tnfa*, which have been proposed to exert opposite effects in several studies. More recently in the Nile tilapia (On), two tnf isoforms, On-*tnfa* and On-*tnfb*, have been identified in a wide range of tissues (96). Experimental infection with *Streptococcus agalactiae* significantly increased *On-tnfa* and *On-tnfb* mRNA levels and Tnf proteins in spleen lymphocytes during the primary response stage of adaptive immunity suggesting the possible involvement of On*-tnfa* and On*-tnfb* in the adaptive immune response of Nile tilapia. Both transcripts increased following activation of spleen lymphocytes by the T cell specific mitogen phytoagglutinine. Finally, recombinant On-Tnfa and On-Tnfb could induce apoptosis of head kidney leukocytes in Nile tilapia, whereby On*-tnfb*, but not On*-tnfa* promoted apoptosis by activating caspase-8 in target cells (96). While both, *tnfa* and *tnfb*, were postulated to be clearly involved as pro-inflammatory cytokines in the lymphocyte-mediated adaptive immune response of Nile tilapia by initiating apoptosis (96), more specific data are required in the future in a comparative approach. So far, Tnfa has been proposed in human patients as potential antagonistic pro-inflammatory cytokine counteracting some beneficial effects of Igf1 and impairing the activity of the Gh/Igf axis, e.g., in neurological and immunological disorders (reviewed by 97, 98).

Whole salmonid fry showed a small upregulation of *igf1* receptor expression during bacterial and viral infections. In particular, *Yersinia ruckeri*-challenge strongly downregulated gene expressions of *igf1* and its receptors in head kidney and spleen and of *igf2* in spleen of adult fish, which highlights a strong repression of IGF-signalling in primary immune tissues (99). The data set is still generally too scarce in light of the important role of fish species in aquaculture and wildlife research. In a study on salinity and the immune health system in Nile tilapia (100), fish reared at 16-ppt salt showed better performance than the population at 20 ppt with a lower mortality and higher expression of ion-regulated genes, stress-related genes in the gills and pro-inflammatory genes *il1b* and interleukin 8 (*il8*) in the liver. Elevated kidney-immune-related genes at 20-ppt salt were proposed to indicate that a higher salinity may predispose the fish to infection and to increased mortality. On the contrary, as proposed by Choi etal. (92) rapid decreases in salinity but not increases lead to immune dysregulation in Nile tilapia. In a recent study in olive flounder (*Paralichthys olivaceus*), after transfer from seawater to freshwater, there was no difference in cortisol levels between active and passive coping flounder while the prolactin level of freshwater-acclimated active coping flounder was higher than of passive acting flounder (101). This underlines the complexity of the orchestration of the neuroendocrine and the immune system.

## Limitations of the study

While this study describes salinity-related changes in genes of the hormones and growth factors in euryhaline tilapia, brain, pituitary, head kidney, and spleen, the amounts of mRNA may not necessarily reflect the amounts of encoded peptides and their activities. Future studies are encouraged to address peptides or activities of hormones and cytokines as well as the osmoregulatory status and immune functions of the fish.

## Conclusion

Our study describes the effects of a bidirectional exposure and acclimation from freshwater to seawater and back to freshwater challenges on the transcripts of several genes associated with osmoregulation and immunity, *igf1*, *igf2*, and *ghr1* in whole brains, pituitaries, spleens and head kidneys as well as the changes in *prl* and *gh* in the pituitaries and *tnfa* expressions in head kidneys and spleens by quantitative PCR in a strongly euryhaline tilapia species. Our results show the influence of salinity-related changes on mRNA contents of several endocrine and immune genes: in brain, 1 or 2 days after seawater and freshwater exposure, respectively, *igf1* and *igf2* genes were decreased. In the pituitary, *prl* gene expression was lowered over the entire seawater period and transiently returned to control levels after a return to freshwater. Gene expressions of *gh* and *igf1* increased immediately after seawater challenge while the *ghr1* gene was lowered after 1 day. In the head kidney, *igf1* was elevated over the entire period, also in freshwater, but in spleen, decreased in seawater and then also increased in freshwater. These results aim at contributing to a more differentiated understanding of the involvement and role of the Gh/Igf-system within the neuro-endocrine and immune organs and the impact of bidirectional saline challenges in organs other than osmoregulatory organs, notably the complex orchestration of growth factors and cytokines.

## Data availability statement

The raw data supporting the conclusions of this article will be made available by the authors, without undue reservation.

## Ethics statement

The animal study was reviewed and approved by CIRAD facilities under the Laboratory agreement for animal experimentation number A-34-172-24.

## Author contributions

J-FB, HD’C, EE, KL and NSh designed the study. GA, J-FB, AC, HD’C, EE, OF, KL, FM, NSe, and NSh performed experiments. J-FB, HD’C, EE, KL, and OR revised the manuscript. All authors contributed to the article and approved the submitted version.

## Funding

Work was supported by the Swiss National Foundation and the Prof. Dr. med. Karl and Rena Theiler-Haag Foundation.

## Acknowledgments

Thanks are due to Manfred Reinecke, Institute of Anatomy, University of Zurich, for the initiation of the original salinity-project. The authors are grateful to Marc Cannone, Aquaculture Unit UPR20, Centre de Coopération Internationale en Recherche Agronomique pour le Développement, Campus International Baillarguet, Montpellier, France for excellent and friendly support.

## Conflict of interest

The authors declare that the research was conducted in the absence of any commercial or financial relationships that could be construed as a potential conflict of interest.

## Publisher’s note

All claims expressed in this article are solely those of the authors and do not necessarily represent those of their affiliated organizations, or those of the publisher, the editors and the reviewers. Any product that may be evaluated in this article, or claim that may be made by its manufacturer, is not guaranteed or endorsed by the publisher.
